# Glutathione S-transferase omega gene polymorphism as a biomarker for human papilloma virus and cervical cancer in Iranian women

**DOI:** 10.4274/jtgga.2018.0056

**Published:** 2018-11-15

**Authors:** Sara Zamani, Amir Sohrabi, Marjan Rahnamaye-Farzami, Seyed Masoud Hosseini

**Affiliations:** 1 Department of Microbiology and Microbial Biotechnology, Shahid Beheshti University Faculty of Life Sciences and Biotechnology, Tehran, Iran; 2 Department of Medical Epidemiology and Biostatistics, Karolinska Institute, Stockholm, Sweden; 3 Research Center of Health Reference Laboratory, Ministry of Health and Medical Education, Tehran, Iran

**Keywords:** Human papilloma virus, cervical cancer, omega gene, polymorphism

## Abstract

**Objective:**

Human papillomavirus (HPV) infection is an important sexually-transmitted infection worldwide. Persistent infections with different high-risk HPV genotypes may cause cervical intraepithelial neoplasia and cervical cancer. Single nucleotide polymorphisms of glutathione S-transferase omega (GSTO) 1 and 2 play an important role in cancer progression. To evaluate GSTO gene polymorphism influence on women’s susceptibility to low-risk or high-risk HPV infections and also risk of cervical cancer development.

**Material and Methods:**

We examined 50 patients with cervical cancer, 43 patients who were positive for HPV, and 43 healthy individuals as negative controls. We used polymerase chain reaction-restriction fragment length polymorphism to determine GSTO1 A140D and GSTO2 N142D variants in study participants.

**Results:**

We found a significant association between the GSTO1 A140D gene polymorphism and HPV 6, 16, 18, 16/18 infections and cervical cancer in Iranian women. We noted a significant difference for the 140AD/142NN combination genotype between patients in the cervical cancer group and healthy controls. There were no significant differences for the GSTO2 N142D genotype and allele frequencies between the patient (i.e., cervical cancer and HPV-positive) groups and controls.

**Conclusion:**

The 140AD genotype, 140D allele, and 140AD/142NN combination genotype seem to confer a protective property in women’s susceptibility to HPV 6, 16, 18, 16/18 infections and cervical cancer. However, the GSTO2 N142D polymorphism is not associated with HPV infections and cervical cancer. It would appear that GSTO1 A140D SNPs likely play a role in the level of susceptibility to HPV-related cervical cancer.

## Introduction

Human papillomavirus (HPV) infections constitute a large portion of sexually-transmitted disease cases worldwide, and up to 70% of sexually active women are infected by HPV during their lifetime. HPVs are divided into high-risk and low-risk genotypes based on their level of association with malignancies (1,2,3,4,5,6).

Generally, 85% of the global burden of HPV infection is occurring in developing countries with high-risk areas such as those in Africa and South America, and North American and Western Asia bear a lower portion of the infection burden (7,8). The variation seen in the occurrence of HPV infection in different regions of the world demonstrates that although HPV is the main cause of cervical cancer, environmental and genetic factors such as genetic polymorphisms also affect the occurrence of this disease (8,9,10).

The human cytosolic glutathione-S-transferase (GST) super family contains at least 16 genes subdivided into eight distinct classes designated as Alpha, kappa, Mu, Omega, Pi, Sigma, Theta, and Zeta. The GST superfamily of antitoxic enzymes can catalyze the conjugation of glutathione to a wide variety of endogenous and exogenous compounds (8,11,12,13) and contribute in many important cellular reactions including the response to environmental stresses, cell proliferation, phase II metabolism, apoptosis, oncogenesis, tumor progression, and drug resistance (8,13). The over expression of these enzymes can induce apoptosis, which can affect cancer development (14). The presence of genetic diversity in this enzymatic super family can affect the antitoxic activities of these enzymes (8). Single nucleotide polymorphisms (SNPs) of this super family can affect the likelihood of cancer development and the chances of success for various treatments (14).

The GST omega (GSTO) class belongs to the GST enzyme super family, which has a cysteine amino acid in its active site. Two actively transcribed GST genes (GSTO1 and GSTO2) are located on the long arm of chromosome 10, and both genes contain 6 exons (13,14,15). 

GSTO members are widely distributed in a range of mammalian tissue types including the liver, colon, heart, ovary, pancreas, prostate, and spleen (13,14). GSTOs have physiologic roles in multidrug resistance, oxidative stress response, and interleukin-1b activation. GSTO genes are polymorphic, and SNPs have been reported in the coding and noncoding regions of these genes. The gene frequency of different substitutions and their effects on enzyme function vary in different populations (14).

The most frequent missense polymorphism in the GSTO1 gene is the Ala140/Asp substitution. This substitution can be found in all populations. The Asp 140 variant has lower thiol transferase activity. The GSTO2 gene is really polymorphic, and 66 SNPs have been reported for this region to date. The most common substitution found across all populations is Asn142/Asp (14,16). SNPs of GSTOs play an important role in cancers such as breast cancer, hepatocellular carcinoma, bile duct carcinoma, urethral cancer, acute lymphoblastic leukemia, and non-small cell lung cancer (13,17).

Recently, there has been great interest in identifying new biomarkers that might provide better results in the earlier recognition of HPV infections and cervical cancer. Currently, no GSTO gene polymorphisms have been explored between HPV infection and genital cancers, but some studies have investigated the interaction between these SNPs and many diseases such as sporadic Alzheimer’s disease, cerebrovascular atherosclerosis, and obstructive pulmonary disease (18).

In this study, we established the frequencies of the GSTO1 and GSTO2 genotypes and allele in an Iranian population. In addition, we investigated whether GSTO gene polymorphisms could influence the risk susceptibility of cervical cancer development in women with HPV genotypes.

## Material and Methods

We collected and evaluated 50 liquid-based cytology (LBC) samples from patients admitted to Mohebe-Yas Hospital in Tehran, Iran, who were diagnosed with cervical intraepithelial neoplasia and cervical cancer. In addition, we also collected and evaluated 43 archived LBC samples from patients with neither cervical cancer nor HPV infection to serve as a negative control comparator, and 43 LBC samples that were positive for HPV genotypes from 2 private laboratories of Tehran, Iran. LBC samples were transferred to the molecular biology department of the health reference laboratory of the Ministry of Health and Medical Education and stored at -20 °C until they were analyzed. The study was approved by the University Ethics Committee. Informed consent was obtained from all subjects. Table 1 presents the demographic clinical data for all patient samples.

### DNA extraction

Genomic DNA was extracted using a High Pure PCR Template Preparation Kit (Roche, Germany). Briefly, according to the manufacturer’s instruction, initially LBCs were lysed, and then DNA binding buffer was added. We mixed them immediately and incubated for 10 min at +70 °C until the cells were digested completely. Isopropanol was added and mixed well. We inserted a High Pure filter tube into one collection tube and transferred the remaining liquid sample with a pipet into upper buffer reservoir of the filter tube. After that, we put the entire High Pure Filter Tube assembly into a standard table-top centrifuge and centrifuged for 1 min at 8000×g. Then, we discarded the flow-through and the collection tube. We combined the filter tube with a new collection tube and added Inhibitor Removal Buffer to the upper reservoir of the filter tube. Centrifugation for 1 min at 8000×g was then performed. Hereafter, the protocol for washing and elution step was accomplished. At the end, micro centrifuge tubes contained the eluted DNA.

### Polymerase chain reaction procedure

In this step, the GSTO1 and GSTO2 genes were amplified using a polymerase chain reaction (PCR) method. These genes were amplified using GSTO1 forward and reverse primers F: 5 ’-GAA CTT GAT GCA CCC TTG GT-3’ and R: 5 ’-TGA TAG CTA GGA GAA ATA ATT AC-3. The primers for GSTO2 were F: 5 ’-AGG CAG AAC AGG AAC TGG AA-3’ and R: 5 ’-GAG GGA CCC CTT TTT GTA CC-3’ (15). The PCR reaction contained 15 μL of Master Mix^®^ 2X (Ampliqon, Denmark) and 1 μL of forward and reverse primers, in which, 15 μL of Master Mix was taken for each sample and mixed with 10 μL of genomic DNA, so that the final mixture volume was 25 μL. PCR cycling was performed with initial denaturation at 94 °C for 5 min, followed by 35 cycles of amplification at 94 °C for 60 seconds, 62 °C for 60 seconds, 72 °C for 60 seconds, and finally at 72 °C for 10 min.

### GSTO1*A140D polymorphism analysis

**Figure 1:**
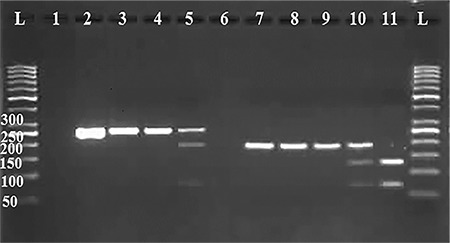
Lanes: (L) 50-bp DNA ladder; (1), Negative Control for GSTO1; (2), PCR product for GSTO1: 254bp fragment; (3 and 4), homozygote AA: 254 bp fragment; (5), heterozygote AD: 254, 186, and 68-bp fragments; (6), Negative Control for GSTO2; (7), PCR product for GSTO2: 185 bp fragment; (8 and 9), homozygote NN: 185-bp fragment; (10), heterozygote ND: 185, 122 and 63 fragments; (11), homozygote DD: 122 and 63 bp fragments; (L) 50-bp DNA ladder PCR: Polymerase chain reaction

For the PCR-restriction fragment length polymorphism (PCR-RFLP) step to indicate C>A transversion polymorphism in exon 4 of the GSTO1 gene, we used the restricting enzyme of CaC8 I (New England BioLabs, USA). The PCR-RFLP mixture included 16 μL of distilled water, 0.7 μL of CaC8 I, three μL of 10X NEBuffer^®^ (New England BioLabs, USA), and 10 μL of PCR products so that the final volume was around 30 μL. This mixture was stored at 37 °C for 1 hour for digestion. The digested products appeared in 3 different patterns: (I) wild-type (140AA) showing 254 fragments; (II) heterozygote (140AD), 68, 186, and 254 bp fragments; and (III) homozygote (140DD) demonstrating 68 and 186 bp fragments (Figure 1).

### GSTO2*N142D polymorphism analysis

The A>G transition polymorphism at codon 142 in exon 4 of GSTO2 was shown via the use of the restricting enzyme MboI (New England BioLabs, USA). The PCR-RFLP mixture for each reaction was similar to that used in the GSTO1*A140D polymorphism analysis. The digested products were shown in 3 patterns: (I) wild-type homozygote (142NN) presenting 185 bp fragments; (II) heterozygote (142ND), 63, 122, and 185 bp fragments; and (III) homozygote (142DD) 122 and 63 fragments. Afterwards, electrophoresis was performed at 100 V for 40 minutes in 1X Tris/Borate/Ethylenediamine tetra acetic acid buffer and 3% agarose gel to detect PCR-RFLP patterns. Products were visualized under ultraviolet light (Figure 1).

### Statistical analysis

We used the IBM SPSS Statistics for Windows, Version 23.0 (Released 2013. Armonk, NY: IBM Corp.) computer software for data analysis. One-way analysis of variance (post hoc, least significant difference method) was used to compare the mean value for age in the different groups employed. The crude and adjusted odds ratio (OR) and 95% confidence intervals (CI) were calculated using binary logistic regression. In addition, Pearson’s chi-square test was used for comparing the relationship between GSTO1 and GSTO2 genotypes and for pathologic staging of CC. A p-value of <0.05 was considered significant. Allele frequencies of GSTO1 and GSTO2 genotype polymorphisms were calculated using the Hardy-Weinberg equilibrium. A chi-square test was employed to study the deviation from the Hardy-Weinberg equilibrium between the observed and expected genotype frequencies in the controls.

## Results

The distribution between the HPV-positive group and controls were not significantly different for age (p=0.679). However, there was a significant difference (p<0.001) in ages between samples in the cervical cancer group and the HPV-negative controls.

The GSTO1 A140D and GSTO2 N142D genotypic frequencies of the HPV-negative control group were in Hardy-Weinberg equilibrium (χ^2^= 1.91 and 0.452, respectively). Allele frequencies in the HPV-negative control population for the GSTO1 gene were 0.825 for the A allele and 0.175 for the D allele. The frequencies for the GSTO2 gene were 0.697 for the N allele and 0.303 for the D allele.

There was a significant difference for the 140 AD genotype and D allele frequency in the cervical cancer group compared with the HPV-negative control group. This indicates a protective property of the AD genotype and D allele. Calculating with binary logistic adjusted for age also revealed a significant difference for the 140AD/142NN combination genotype (p=0.016) with a protective function for this genotype in these groups. The OR analysis and 95% CI between the HPV-positive group and HPV-negative controls were not significantly different for genotypes and allele frequencies (Table 2).

HPV 16, HPV 18, and HPV 6 were the most prevalent subtypes in the cervical cancer group and the HPV-positive group. The sum of patients with HPV 16, HPV 18, HPV 16/18, and HPV 6 infections in the cervical cancer group and the HPV-positive groups were 41, 32, 21, and 23, respectively. The details of HPV genotyping results are not shown in this study. The relationship between these patients and those in the HPV-negative control group for these genotypes were calculated using binary logistic adjusted for age, like in the previous analysis.

In individuals positive for HPV 16, the frequencies of GSTO1 genotypes were 38 for AA, 3 for AD, and 0 for DD. This analysis revealed a significant protective attribute for the AD genotype [OR= 0.075; 95% CI: (0.015 to 0.386); p=0.002]. The frequencies of GSTO2 genotypes were 18 for NN, 19 for ND, 4 for DD, and 23 for ND/DD. We found no significant difference for GSTO2 N142D between the HPV 16-positive group and the HPV-negative controls (p>0.05). The GSTO1 A140D and GSTO2 N142D combination genotype frequencies were 16 for 140AA/142NN, 18 for 140AA/142ND, 4 for 140AA/142DD, 2 for 140AD/142NN, and 1 for 140AD/142ND. There was a significant difference in the 140AD/142NN combination genotype between patients positive for HPV 16 and those in the HPV-negative group [OR= 0.058; 95% CI: (0.007 to 0.503); p=0.01] with the protective role. There were no significant differences for other combination genotypes between patients positive for HPV 16 and those in the HPV-negative group (p>0.05). 

For patients positive for HPV 18, the frequencies of GSTO1 genotypes were 28 for AA, 4 for AD, and 0 for DD. This analysis showed a significant protective role for the AD genotype [OR= 0.113; 95% CI: (0.024 to 0.525); p=0.005]. The frequencies of the GSTO2 genotypes were13 for NN, 18 for ND, 1 for DD, and 19 for ND/DD. We found no significant difference for GSTO2 N142D between HPV 18-positive samples and the HPV-negative control samples (p>0.05). The frequencies of GSTO1 A140D and GSTO2 N142D combination genotype were 11 for 140AA/142NN, 16 for 140AA/142ND, 1 for 140AA/142DD, 2 for 140AD/142NN, 2 for 140AD/142ND, and 0 for 140AD/142DD. There was a significant difference for the 140AD/142NN combination genotype between patients positive for HPV 18 and those in the HPV-negative group [OR= 0.058; 95% CI: (0.006 to 0.532); p=0.012] with the protective role. There were no significant differences for other combination genotypes between patients positive for HPV 18 and those in the HPV-negative group (p>0.05).

In the HPV 16/18 co-infection group, the frequencies of GSTO1 genotypes were 19 for AA, 2 for AD, and 0 for DD. The analysis indicated a significant protective role for the AD genotype [OR= 0.055; 95% CI: (0.006 to 0.462); p=0.008]. The frequencies of GSTO2 genotypes were 8 for NN, 12 for ND, 1 for DD, and 13 for ND/DD. We found no significant difference for GSTO2 N142D between the HPV 16/18 co-infection group samples and the HPV-negative controls (p>0.05). The GSTO1 A140D and GSTO2 N142D combination genotype frequencies were 7 for 140AA/142NN, 11 for 140AA/142ND, 1 for 140AA/142DD, 1 for 140AD/142NN, and 1 for 140AD/142ND. There was a significant difference in the protective role for the 140AD/142NN genotype between HPV 16/18-positive patients and HPV-negative controls [OR= 0.018; 95% CI: (0.01 to 0.403); p=0.011]. There was no significant difference for other combination genotypes between HPV 16/18-positive patients and HPV-negative controls (p>0.05).

In the samples positive for HPV 6, the frequencies of GSTO1 genotypes were 22 for AA, 1 for AD, and 0 for DD. This analysis showed a significant protective role for the AD genotype [OR= 0.056; 95% CI: (0.006 to 0.519); p=0.011]. The frequencies of GSTO2 genotypes were 10 for NN, 11 for ND, 2 for DD, and 13 for ND/DD. We found no significant difference for GSTO2 N142D between the HPV 6-positive patients and the HPV-negative controls (p>0.05). The frequencies of GSTO1 A140D and GSTO2 N142D combination genotypes were 9 for 140AA/142NN, 11 for 140AA/142ND, 2 for 140AA/142DD, and 1 for 140AD/142NN. We found no significant difference for these combination genotypes between HPV 6-positive patients and HPV-negative controls (p>0.05), although the result of the analysis for 140AD/142NN genotype was an OR of 0.094, with 95% CI of 0.008 to 1.06 (p=0.056). Therefore, although there was an association between this genotype and HPV6 infection, the association was not significant.

There was no significant association between GSTO1 and GSTO2 genotypes and pathologic staging of cervical cancer (Table 3). All patients with cervical intraepithelial neoplasia (CIN) grade I, II, and more than 82% of patients with CIN grade III and invasive cervical cancer were recognized with the 140AA genotype, and none were recognized with the 140DD and 142DD genotypes.

GSTO1 and GSTO2 genotype frequencies in individuals with HPV 11, 26, 31, 33, 35, 39, 45, 51, 52, 53, 54, 56, 58, 59, 61, 66, 68, 70, 82, and 89 infections are summarized in Table 4.

## Discussion

This study investigated the association between GSTO1 A140D and GSTO2 N142D polymorphisms and susceptibility to HPV infection and cervical cancer progression in Iranian women. To the best of our knowledge, no other previous study has investigated this issue in Iranian populations. Cervical cancer is one of the more prevalent causes of death among women, leading to approximately 270,000 deaths annually (2,19,20). Persistent genital infections with different high-risk HPV genotypes, specifically HPV 16 and 18, lead to CIN and cervical cancer (1,21,22,23). It would seem that investigations should be performed on the recognition and development of genetic and epigenetic patterns as molecular prognostic biomarkers in the diagnosis of early stage of cervical cancer (15,24). 

The GSTO enzyme is a new class in the GST super family which conjugated glutathione to electrophilic intermediates and detoxifies endogenous and exogenous compounds (15,25,26). GSTO1 is expressed in a wide range of human tissues and plays a role in apoptosis. This enzyme is a potential source of intracellular glutathione, which protects against cellular oxidative stresses (16). This protective aspect against cell toxicity may be weakened if enzymatic activity is reduced. Some studies showed a significant reduction in thiol transferal activity resulting from aspartic acid substitution, whereas other studies found no significant reduction in the enzyme activity (16). The allele frequency of the GSTO1 A140D polymorphism in our study was similar to that reported by Ada et al. (16) in a Chinese population study that compared European, American, and other Asian populations.

The GSTO2 enzyme shows a new type of activity that is not seen in other GSTs including glutathione-dependent thiol transferase, monomethyl arsenate reductase, and dehydroascorbate reductase activity. The gene encoding GSTO2 is a polymorphic gene with a single nucleotide polymorphism causing an asn142Asp (N142D) substitution. This substitution may alter the function of the GSTO2 enzyme (15,17,27). The allele frequencies of the GSTO2 N142D polymorphism in our study are similar to those reported by Rezazadeh et al. (15) in comparison with Italy, Thailand, Japan, and Turkey.

Based on our findings, the 140AD genotype, 140D allele, and 140AD/142NN combination genotype seem to confer a protective property in women’s susceptibility to HPV 6, 16, 18 and 16/18 infections and cervical cancer. To the best of our knowledge, there have been no investigations to determine association between the GSTO1 A140D and GSTO2 N142D and HPV infections or cervical cancer. However, Sanguansin et al. (18) suggested that the GSTO1*D140 variant genotype might play a protective role against head and neck cancer in the Thai population. In contrast, Ada et al. reported no significant association between the GSTO1 A140D polymorphism and susceptibility to non-small cell lung cancer in the Turkish population (16). Also, Rezazadeh et al. found that the frequency of GSTO1 A140D polymorphism was not associated with childhood pre-B acute lymphoblastic leukemia in the Iranian population (15). 

In our study, the GSTO2 N142D polymorphism was not associated with HPV infections and cervical cancer. Sanguansin et al. (18) revealed that the frequency of GSTO2 genotype was not significantly different between patients with head and neck cancer and controls in the Thai population, which is in agreement with our results. Also, Rezazadeh et al. (15) showed that there was no significant association between pre-B acute lymphoblastic leukemia and GSTO2 N142D polymorphism in the Iranian population. However, Khosravi et al. (17) demonstrated that individuals with DD genotype were more susceptible to developing hepatic failure leading to liver transplantation. 

Therefore, we suggest that GSTO1 A140D gene polymorphisms likely play an inconspicuous role in the level of susceptibility to HPV-related cervical cancer. Future studies with a larger number of patients should explore the additional effect of these polymorphisms with other HPV infections or cervical cancer risk factors. 

Our study was limited by the relatively small number of patients evaluated. The clinical dataset from the subjects was another limitation of the study. Some important risk factors like smoking status, which have a critical role in GSTO polymorphism interactions and HPV infections or cervical cancer were missed because our patients were not new (they were collected in 2012), and we did not have more information from the subjects. In addition, approximately 65% of participants in the HPV-positive group were infected with low-risk HPV genotypes (HPV 6, n=16; other low-risk HPV types, n=2). 

In conclusion, future investigations should be performed on larger groups of participants, especially on women with high-risk HPV genotypes and other sexually-transmitted infections in order to find any association between SNPs and cervical malignancies in developing countries. Cancer screening, particularly early diagnosis in the first stages can be helpful in national health programs using an approved molecular biomarker.

## Figures and Tables

**Table 1:**
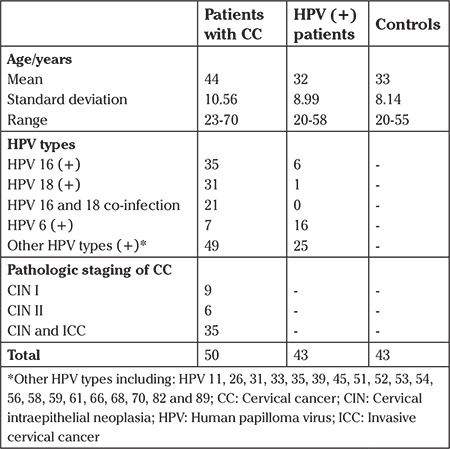
Demographic clinical data of subjects

**Table 2:**
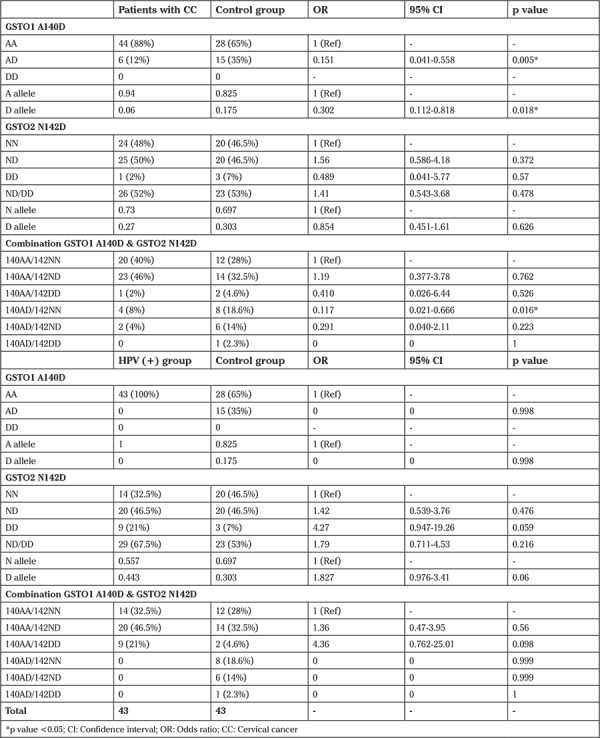
Calculated OR and 95% CI with binary logistic regression and adjusted for age population study

**Table 3:**
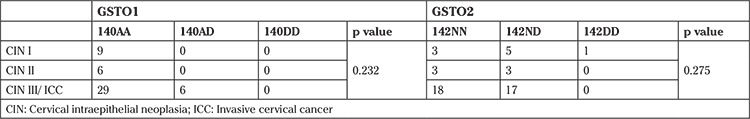
Relationship between GSTO1 and GSTO2 genotypes and pathologic staging of patients with cervical cancer

**Table 4:**
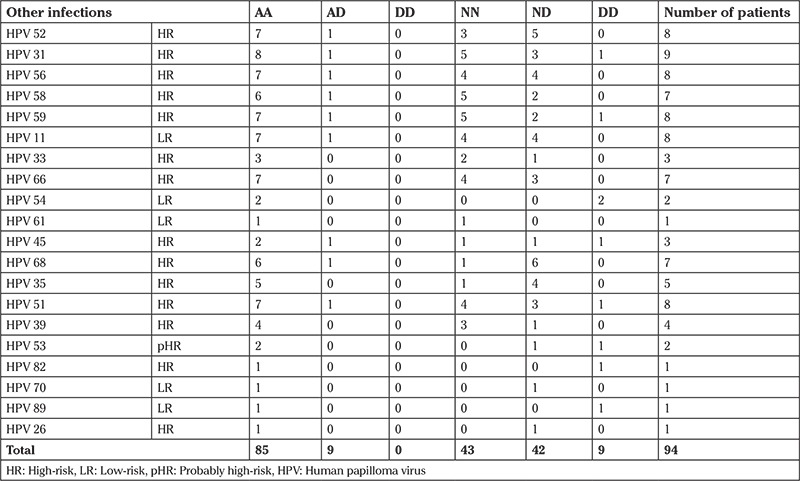
GSTO1 and GSTO2 genotype frequency in women with HPV genotype infections

## References

[1] Bharti AC, Shukla S, Mahata S, Hedau S, Das BC (2009). Anti-human papillomavirus therapeutics: facts & future. Indian J Med Res.

[2] Jia H, Wang X, Long Z, Li L (2015). Human papillomavirus infection and cervical dysplasia in female sex workers in Northeast China: an observational study. BMC Public Health.

[3] Pour BT, Hosseini SM, Kardi MT (2017). Frequency of human papilloma virus genotypes in patient samples from Isfahan (Iran) using polymerase chain reaction reverse dot blot method. Jundishapur J Microbiol.

[4] Shayanfar N, Babaheidarian P, Rahmani H, Azadmanesh K, Sohrabi A, Mohammadpour M, et al (2012). Epidermodysplasia verruciformis associated with plasmablastic lymphoma and hepatitis B virus infection. Acta Dermatovenerol Croat.

[5] Shayanfar N, Hosseini N, Panahi M, Azadmanesh K, Mohammad pour M, Kadivar M, et al (2013). Detection of mucosal type human papillomavirus in cutaneous squamous cell carcinoma in Iran. Pathol Res Pract.

[6] Sohrabi A, Hajia M, Jamali F, Kharazi F (2017). Is incidence of multiple HPV genotypes rising in genital infections?. J Infect Public Health.

[7] Wang D, Wang B, Zhai JX, Liu DW, Sun GG (2011). Glutathione S-transferase Ml and T1 polymorphisms and cervical cancer risk: A meta-analysis. Neoplasma.

[8] Zhen S, Hu CM, Bian LH (2013). Glutathione S-transferase polymorphism interactions with smoking status and HPV infection in cervical cancer risk: an evidence-based meta-analysis. PLoS One.

[9] Gao LB, Pan XM, Li LJ, Liang WB, Bai P, Rao L, et al (2011). Null genotypes of GSTM1 and GSTT1 contribute to risk of cervical neoplasia: an evidence-based meta-analysis. PLoS One.

[10] Settheetham-Ishida W, Yuenyao P, Kularbkaew C, Settheetham D, Ishida T (2009). Glutathione S-transferase (GSTM1 and GSTT1) polymorphisms in cervical cancer in Northeastern Thailand. Asian Pac J Cancer Prev.

[11] Coughlin SS, Hall IJ (2002). Glutathione S-transferase polymorphisms and risk of ovarian cancer: A HuGE review. Genet Med.

[12] Hasan S, Hameed A, Saleem S, Shahid SM, Haider G, Azhar A (2015). The association of GSTM1 and GSTT1 polymorphisms with squamous cell carcinoma of cervix in Pakistan. Tumour Biol.

[13] Xu YT, Wang J, Yin R, Qiu MT, Xu L, Wang J, et al (2014). Genetic polymorphisms in Glutathione S-transferase Omega (GSTO) and cancer risk: a meta-analysis of 20 studies. Sci Rep.

[14] Board PG (2011). The omega-class glutathione transferases: structure, function, and genetics. Drug Metab Rev.

[15] Rezazadeh D, Moradi MT, Kazemi A, Mansouri K (2015). Childhood Pre-B acute lymphoblastic leukemia and glutathione S-transferase omega 1 and 2 polymorphisms. Int J Lab Hematol.

[16] Ada TG, Ada AO, Kunak SC, Alpar S, Gulhan M, Iscan M (2013). Association between glutathione S-transferase omega 1 A140D polymorphism in the Turkish population and susceptibility to non-small cell lung cancer. Arh Hig Rada Toksikol.

[17] Khosravi M, Saadat I, Karimi MH, Geramizadeh B, Saadat M (2013). Glutathione S-transferase omega 2 genetic polymorphism and risk of hepatic failure that lead to liver transplantation in Iranian population. Int J Organ Transpl Med.

[18] Sanguansin S, Petmitr S, O-Charoenrat P, Pongstaporn W (2012). Association of glutathione S-transferase omega gene polymorphisms with progression of head and neck cancer. Mol Biol Rep.

[19] Sohrabi A, Hajia M (2017). Cervical cancer and genital infections: assessment of performance and validation in human papillomavirus genotyping assays in Iran, its neighboring countries and Persian Gulf area. Iran J Pathol.

[20] Sohrabi A, Mirab-Samiee S, Modarresi MH, Izadimood N, Azadmanesh K, Rahnamaye-Farzami M (2014). Development of in-house multiplex real time PCR for human papillomavirus genotyping in Iranian women with cervical cancer and cervical intraepithelial neoplasia. Asian Pac J Cancer Prev.

[21] Sohrabi A, Farzami R, Samiee SM, Modarresi MH (2015). An overview on papillomaviruses as the main cause of cervical cancer, Iran. J Obstet Gynecol Infertil.

[22] Sohrabi A, Rahnamaye-Farzami M, Mirab-Samiee S, Mahdavi S, Babaei M (2016). Comparison of in-house multiplex real time PCR, Diagcor Geno Flow HPV Array test and INNO-LiPA HPV genotyping extra assays with LCD-Array Kit for human papillomavirus genotyping in cervical liquid based cytology specimens and genital lesions in Tehran, Iran. Clin Lab.

[23] Hajia M, Sohrabi A (2018). Possible Synergistic Interactions among Multiple HPV Genotypes in Women Suffering from Genital Neoplasia. Asian Pac J Cancer Prev.

[24] Sohrabi A, Mirab-Samiee S, Rahnamaye-Farzami M, Rafizadeh M, Akhavan S, Hashemi-Bahremani M, et al (2014). C13orf18 and C1orf166 (MULAN) DNA genes methylation are not associated with cervical cancer and precancerous lesions of human papillomavirus genotypes in Iranian women. Asian Pac J Cancer Prev.

[25] Ciba M, AK M, Karahalil B (2016). Alpha-glutathione-s-transferase can be a biomarker for both drug-related toxicity as well as individual susceptibility. Minerva Psichiatrica.

[26] Helzlsouer KJ, Selmin O, Huang HY, Strickland PT, Hoffman S, Alberg AJ, et al (1998). Association between glutathione S-transferase M1, P1, and T1 genetic polymorphisms and development of breast cancer. J Natl Cancer Inst.

[27] Saadat M (2012). Genetic polymorphism of N142D GSTO2 and susceptibility to breast cancer: a meta-analysis. Mol Biol Res Commun.

